# New York Post-Graduate Medical School and Hospital

**Published:** 1908-09

**Authors:** 


### New York Post-Graduate Medical School and Hospital

IN celebration of the twenty-fifth anniversary of the founding of this, the first institution for the teaching of graduates in medicine established in this country, the faculty has issued a volume entitled, Contributions to the Science of Medicine and Surgery. It is an imperial octavo volume of 485 pages, printed on calendered deckle-edged paper, with wide margins and bound in illuminated dark green paper:

An introduction to the book is a biographical sketch of Dr. Daniel Bennett St. John Roosa, the founder of the school and president of the faculty from the beginning. A full-page etching of Dr. Roosa, in portrait, constitutes an imposing frontispiece. The sketch of his life is written by Dr. Charles L. Dana and is a concise history of the busy life of this remarkable man. In the course of the sketch Dr. Dana quotes from the article written by Dr. Maurice J. Lewi, secretary of the state board of medical examiners, published in the *Post-Graduate* last April, in which Dr. Lewi said:

Ever fighting for higher standards, ever striving to advance the interests of the profession, always on the alert for methods and means of inciting the ambition of those teaching the student body, he was a welcome addition to all councils where these points were under advisement.

As indicative of the multitudinous topics which engaged Dr. Roosa's attention and which in turn were presented to the legislature of the state of New York, either directly by him or through the committee of which he was chairman, (see reports Committee on Legislature, 1890 and 1891, *Transactions* of the Medical Society of the State of New York), the following may be quoted:

1. The transfer of the chronic insane from county asylums to state institutions.
2. "An act to prevent blindness."
3. The regulation and the control of the embalming process.
4. Advancing the standard of academic requirements for all persons entering upon a career of medicine.
5. Safeguarding the interests of the New York state medical practitioners as against practitioners resident in other states, desirous of being admitted to practise in New York state.

Dr. Roosa ceased his official connection with the state society many years ago, but his interest in the welfare of the medical profession never lagged. He was ever ready to come to the rescue when danger threatened, and many a time, at a personal loss of income from his large practice, he went to Albany and to even greater distances to exert his voice and efforts for or against measures which had a bearing upon the well-being of medical practice.

In the biographic sketch Dr. Dana points out that Dr. Roosa was noted for his skill and leadership in the topics of his specialties, ophthalmology and otology, to his success as a teacher, and for his activity in advancing the interest of medical education.

The body of the volume consists of forty-seven selected papers written by as many members of the faculty and which constitute a distinct contribution to the science of medicine and surgery. The book is a great credit to those concerned, not excepting the editor, Dr. Henry T. Brooks.

